# Groove Pancreatitis in Focus: Tumor-Mimicking Phenotype, Diagnosis, and Management Insights

**DOI:** 10.3390/jcm14051627

**Published:** 2025-02-27

**Authors:** Marina Balaban, Daniel Vasile Balaban, George Mănucu, Săndica Nicoleta Bucurică, Raluca Simona Costache, Florentina Ioniță-Radu, Mariana Jinga, Cristian Gheorghe

**Affiliations:** 1 Doctoral School, Carol Davila University of Medicine and Pharmacy, 020021 Bucharest, Romania; 2 Internal Medicine and Gastroenterology Department, Carol Davila University of Medicine and Pharmacy, 020021 Bucharest, Romania; 3 Gastroenterology Department, Central Military Emergency University Hospital, 010825 Bucharest, Romania; 4 Radiology Department, Central Military Emergency University Hospital, 010825 Bucharest, Romania; 5 Gastroenterology Department, Fundeni Clinical Institute, 022328 Bucharest, Romania

**Keywords:** groove pancreatitis, chronic pancreatitis, tumor-like, groove carcinoma

## Abstract

****Background/Objectives**:** Groove pancreatitis (GP) is an uncommon pancreatic condition implying a challenging differential diagnosis. This study aims to comprehensively evaluate the main risk factors, clinical presentation, imaging and endoscopic characteristics of patients with GP, providing insights into an effective diagnostic approach and therapeutic strategies. **Methods***:* A retrospective analysis was conducted on patients diagnosed with GP, with demographic and clinical data collected. The diagnostic route was followed by an upper endoscopy and was finally confirmed by cross-sectional imaging. In patients with high malignancy suspicion or with an uncertain diagnosis, a pancreatic endoscopic ultrasound (EUS) was further performed. According to imaging features, we divided patients into two categories: with and without tumor-like appearance. **Results***:* Altogether, 23 patients were included, 11 in the tumor-like category, and 12 in the non-tumor-like group; 95.6% were men, 78.2% alcohol consumers, and 73.9% smokers. In both groups, the main symptom was abdominal pain, followed by nausea and vomiting. The most frequent finding at upper endoscopy was edematous duodenal mucosa (16 patients, 80%), followed by mucosal hyperemia (8 patients, 40%). The main finding at cross-sectional imaging was duodenal wall thickening (14 patients, 60.9%), followed by pancreatic head enlargement and duodenal wall cysts (both seen in 12 patients, 52.2%). The EUS predominantly showed duodenal wall thickening (13 patients, 68.4%), and intramural and paraduodenal cysts (10 patients, 52.6%). **Conclusions***:* GP predominantly affects men with a history of chronic alcohol and tobacco use. Its primary diagnostic challenge lies in distinguishing it from pancreatic carcinoma, with an accurate diagnostic workup being crucial in clinical practice.

## 1. Introduction

Groove pancreatitis (GP) is a rare, focal form of chronic pancreatitis (CP), affecting the area between the duodenum, the pancreas, and the common bile duct (CBD). The term “groove pancreatitis”, together with “cystic dystrophy of duodenal wall” and “cystic dystrophy on aberrant pancreas of the duodenal wall” are all covered by the general term “paraduodenal pancreatitis”. Since 1982, it has been classified in two types: the *pure type* (involving only the groove area) and the *segmental type* (inflammation in the groove area, but also extended to the pancreatic head) [1]; a *diffuse type* has also been reported, in which the groove inflammation extends beyond the cephalopancreatic area, involving the entire gland and creating the picture of CP, regarded as CP with groove involvement [2,3]. From a pathological point of view, it can be classified as cyst-forming type, mass-forming type (with a solid tumoral appearance), or a combination solid–cystic type. It predominantly affects men more than women, with smoking and alcohol consumption being the primary risk factors associated with GP [3].

The main challenge in clinical practice is the differential diagnosis of GP because of its pseudotumoral aspect. At presentation, it can be easily misdiagnosed as pancreatic malignancy or autoimmune pancreatitis [4]. Conversely, excluding neoplasia in a patient with features of GP can pose diagnostic difficulties. Differentiating GP from malignancy is particularly challenging in patients with the solid pathological form of GP, compared to those with cystic form. Common diagnostic approaches include cross-sectional imaging techniques, such as computed tomography (CT) and magnetic resonance imaging (MRI), along with biological and clinical features. Endoscopic ultrasound (EUS) is frequently required for confirmation, as it remains the most sensitive modality for diagnosing GP [3]. Abdominal pain remains the most common symptom in patients with GP, often accompanied by signs of upper digestive obstruction, including nausea, vomiting, and weight loss. Less commonly reported symptoms include jaundice, diarrhea, and steatorrhea [5,6,7]. Initial management is typically conservative, including pancreatic rest, along with alcohol and tobacco cessation. Symptomatic treatment varies based on individual symptoms and may include pain relief measures, antispasmodics, neuromodulators, and muscle relaxants. Long-acting somatostatin analogs can also be utilized, and in cases where pain remains uncontrolled, narcotic analgesics are ultimately considered [8]. Therapeutic management also includes endoscopic treatment, which is reserved for patients with pancreatic or biliary duct strictures, pancreatic fluid collections or duodenal obstruction [9]. Finally, surgery becomes the therapeutic option for patients who fail to respond to conservative and endoscopic therapies, or when diagnostic doubts persist with a high suspicion of malignancy and equivocal preoperative workup [8].

Despite advancements in diagnostic tools, increased knowledge and awareness of this pathology, GP remains an infrequent diagnosis in clinical practice among CP patients, with only small series and cohorts published so far. Our aim was to analyze clinical, imaging, and endoscopic features in a cohort of GP patients and provide a proposal for the diagnostic workup for these patients.

## 2. Materials and Methods

### 2.1. Study Design

This is a retrospective study that included all patients presented to Carol Davila Central Military Emergency University Hospital Bucharest between 1 January 2018 and 30 June 2024 that had a diagnosis of GP after a complete diagnostic workup. The diagnosis was established based on demographic, clinical, and laboratory features and confirmed by cross-sectional imaging, upon the presence of typical features of GP.

### 2.2. Participants

The patients included were required to have the following inclusion criteria: adults over the age of 18, diagnosed with GP according to CT or MRI imaging. At the decision of the treating physician, some of the patients were also referred for EUS examination, either for consolidating the diagnosis, excluding differential diagnoses or tissue acquisition. In patients with the suspicion of malignancy on the background of pancreatitis, EUS-guided tissue sampling was carried out according to ESGE guidelines [10]. We excluded patients with CP without the typical findings of GP. We also excluded patients initially diagnosed with GP according to the imaging aspect, whose histopathological results on tissue sampling were in favor of malignancy (two patients, one with pancreatic adenocarcinoma and one with lymphoma).

### 2.3. Definitions

The term “groove” refers to the area between the head of the pancreas, the wall of the duodenum, and the distal CBD. The imaging features in favor of inflammation in the groove area, and thus the diagnosis of GP, were those described in the literature: a low enhancing hypodense area in the pancreatoduodenal groove together with thickening of the duodenal wall and paraduodenal cysts at CT scan, while at MRI, a hypointense mass on T1-weighted images and an isointense or mildly hyperintense area on T2-weighted images together with focal thickening of the second part of the duodenum, abnormally increased enhancement of the descending duodenum, and cystic changes in the area of the dorsal pancreatic duct [11,12,13]. EUS descriptors include thickness of the duodenal wall and the presence of intramural or paraduodenal cysts or mass-forming lesions in the groove area. A tumor-like pattern was noted from CT, MRI, or EUS results. The term “tumor-like” is illustrative for the presence of a solid mass in the background of pancreatitis, commonly seen in the setting of mass-forming chronic pancreatitis. This has been highlighted particularly in the solid form of GP, which can mimic pancreatic malignancy [14]. The data on CBD and main pancreatic duct (MPD) caliber were also collected.

Management includes a three-tier approach: conservative measures, endoscopic interventions, and surgery. The conservative treatment included all of the non-invasive actions taken once the diagnosis was made, starting with pancreatic rest, analgesics and other symptomatic medications, cessation of alcohol and smoking, and recommendations for a healthy diet [12]. By endoscopic treatment, we referred to all measures taken endoscopically in order to treat GP-associated symptoms or complications. Such measures include cyst aspiration/drainage, pancreatic or biliary stent placement, including stenting of minor papilla, and duodenal dilatation [15]. Surgical treatment was considered any surgical intervention performed in order to alleviate the patient’s symptoms or to treat the complications of groove area inflammation.

### 2.4. Laboratory Parameters

Fasting serum glucose levels were assessed on venous blood samples. Hyperglycemia was considered as a fasting serum glucose more than or equal to 100 mg/dL. The carbohydrate antigen 19-9 (CA19-9) is a tumor marker primarily associated with pancreatic cancer, although it can also be elevated in other conditions, both benign and malignant [16]. Determination of CA19-9 levels was performed on venous blood samples, with values greater than 37 U/mL being considered as elevated. The serum albumin level, as a marker of patients’ nutritional status, was performed on venous blood samples. The levels less than 3.5 g/dL were considered as hypoalbuminemia.

### 2.5. Data Analysis

Figures, tables, and graphs were generated using Microsoft Excel version 16.77, Microsoft Word version 16.35, and the BioRender.com platform. The continuous variables were expressed by median (IQR) and categorical variables by number (%).

## 3. Results

Altogether 23 patients with GP were included in our study, with a median age of 56 years and 95.6% (22 patients) males. This represents a small proportion compared to the total number of admissions for acute (*n* = 615) and chronic pancreatitis (*n* = 115) during the time period of the study. The median follow-up period for our GP cases was 18 months after first presentation. Four patients (17.4%) died during follow-up—two from cardiovascular disease, one from pulmonary malignancy, and one from severe alcoholic hepatitis.

As one of the main diagnostic challenges of GP is pancreatic malignancy, we split patients into two categories: those with and those without a tumor-like appearance. Nine patients (39.1%) had an imaging description of a “tumor-like” mass at CT/MRI; another 2 patients had a tumor-like aspect at EUS examination, not described at cross-sectional imaging. Conversely, two patients who were initially thought to be GP were finally diagnosed with gastrointestinal malignancies and excluded from analysis.

The patient characteristics among the two subgroups, including demographics, prevalence of risk factors, clinical features, and therapies, are summarized in Table 1.

### 3.1. Risk Factors

**Figure 1:**
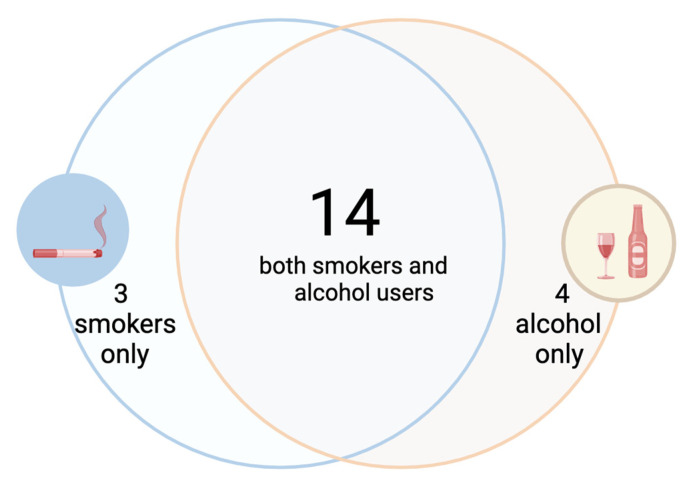
The distribution of alcohol consumption and smoking in the study lot (created with BioRender.com).

Eighteen patients (78.2%) declared a history of chronic alcohol consumption. The number of active smokers was 17 (73.9%). Only 2 patients had no history of smoking or alcoholism (Figure 1).

### 3.2. Clinical Features and Laboratory Workup

**Figure 2:**
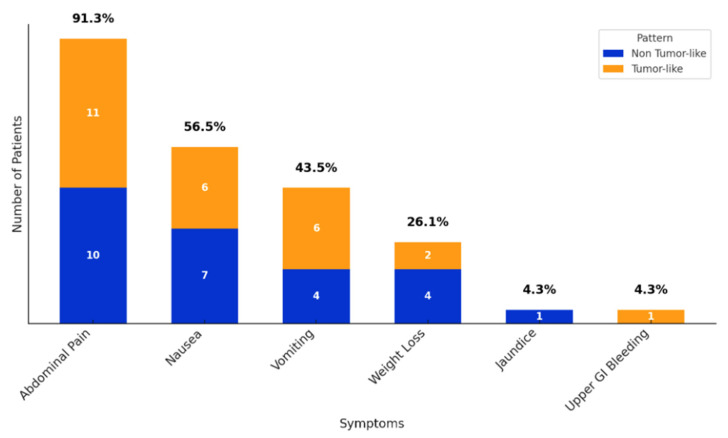
Distribution of patients’ symptoms according to the tumor-like pattern.

The main symptom at presentation was abdominal pain, which was seen in almost all patients, followed by nausea and vomiting, reflecting the obstructive effect of the groove inflammation. One in four patients reported weight loss. Jaundice was rarely seen as the main presenting symptom in GP. An atypical symptom at presentation was upper gastrointestinal bleeding, seen in one patient—shown in Figure 2.

With regard to glycemic dysregulation, which is commonly seen in exocrine pancreatic diseases, hyperglycemia was seen in 13/23 patients (56.5%), with only 2 of them having pre-existing DM. Among the entire cohort, 3 patients (13%) had previously long-standing DM.

Concerning the CA19-9 level, elevated values were found in 4/23 (17.39%) patients. One of these patients was also diagnosed with lung cancer and died during the follow-up. The other 3 patients had CA19-9 levels up to 3 times the upper limit of normal, without associated hyperbilirubinemia or jaundice, without increase during follow-up, and without evidence of tumor-like pattern at cross-sectional imaging.

As an indicator for nutrition status, the serum albumin level was noted in 20/23 patients, with 3 of them (15%) having hypoalbuminemia.

### 3.3. Upper Endoscopy Findings

**Figure 3:**
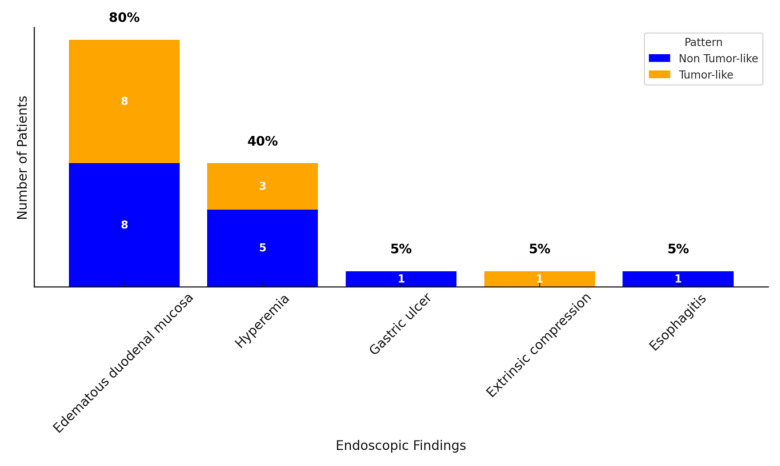
Upper endoscopy findings in patients with GP according to the tumor-like pattern.

An upper endoscopy was performed in 20 out of 23 patients. The main finding was edematous duodenal mucosa with luminal narrowing that could be passed with the scope. In addition to the inflammatory stenosis of the duodenal mucosa, an aspect of extrinsic compression was reported in one patient. Associated gastric hyperemia, esophagitis, and gastric ulcer were also reported in our patients with GP—shown in Figure 3.

### 3.4. Imaging Findings

**Figure 4:**
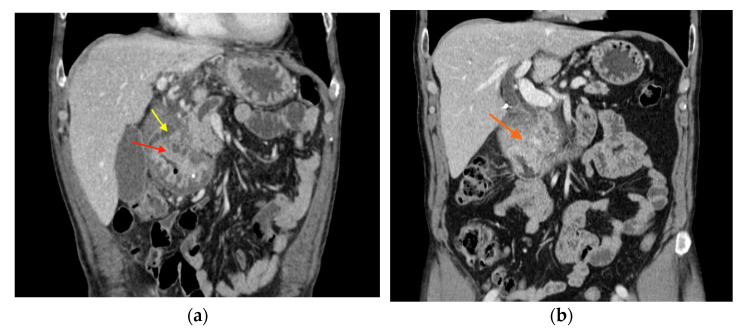
(**a**) CT image in a patient with non-tumor-like GP (yellow arrow: intramural cysts; red arrow: duodenal wall thickening). (**b**) CT image in a patient with tumor-like GP (orange arrow: tumor-like aspect of the head of the pancreas).

All patients had been evaluated by a cross-sectional imaging method, with a CT scan being performed in 15 patients (65.2%) and MRI in 8 patients (34.8%). The pathological findings are listed in Table 2. We noted the duodenal wall thickening as the most frequently described feature (60.9%), followed by pancreatic head enlargement (52.2%) and the presence of paraduodenal and duodenal wall cysts (52.2%)—shown in Figure 4. Importantly, the tumor-like aspect was described in more than a third of the patients (39.1%)—shown in Table 2.

Anatomical anomalies of the pancreas were noted in one patient in our cohort. The MRI revealed annulare pancreas in this patient, together with features such as duodenal wall thickening, pancreatic head enlargement, duodenal wall cysts, and Wirsung dilatation.

### 3.5. Pancreatic EUS

**Figure 5:**
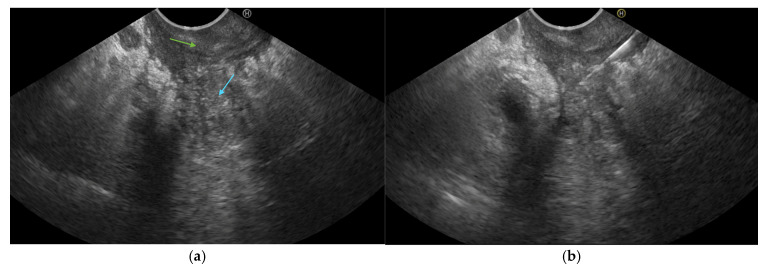
(**a**) EUS image in a patient with tumor-like aspect of GP (green arrow: duodenal wall thickening; blue arrow: tumor-like aspect). (**b**) EUS-FNA in a patient with tumor-like aspect of GP.

In 19/23 patients, a pancreatic EUS was performed. The pancreas evaluation was made by both trans-gastric and trans-duodenal approach in 15 patients, while in 4 patients, because of the luminal narrowing that did not allow passage of the scope, the examination was performed only by gastric view. In 7 patients, an EUS puncture was performed because of malignancy suspicion. FNA was done in 3 patients, while FNB was done in 4 patients, reflecting a change in practice from FNA to FNB in our service during the studied time period (Figure 5b).

The most frequently described feature in EUS was also the duodenal wall thickening (68.4%), followed by intramural and paraduodenal cysts (52.6%). The tumor-like aspect was noted in 31.6% of the patients—shown in Table 3 and Figure 5a.

### 3.6. Management

Most of the patients were treated conservatively, including pancreatic rest, pain control, and cessation of smoking or alcohol consumption. An endoscopic treatment was performed in 2 patients, one cyst drainage and one biliary stent placement. A surgical treatment was performed in 4 patients. The surgical interventions were as follows: gastroenteroanastomosis (in 3 patients), and pseudocystojejunostomy (1 patient)—shown in Table 4. Three of the 4 patients referred to surgery had a long-term disease (between 21 and 46 months).

## 4. Discussion

Although it is considered a rare form of CP, GP is diagnosed in around 20% of patients undergoing pancreaticoduodenectomy for CP [17]. Among the risk factors for GP occurrence, there is a strong correlation with smoking and alcohol consumption. In addition to these traditional factors associated with pancreatic injury [18], there are several other factors that contribute to development of GP.

### 4.1. Pathogenesis

The main factor involved in the pathogenesis of groove pancreatitis is thought to be the impaired pancreatic juice outflow. First of all, anatomical or functional obstruction of the minor papilla is implied. The obstruction of minor papilla is due to inflammation or anatomical changes in the duodenum. The secretions will thus be redirected to the main pancreatic duct at an acute angle, leading to an incomplete drainage in the head of the pancreas, increased intraductal pressure, formation of fluid collections, and leakage of the secretions into the duodenal groove [19].

GP may also result from the obstruction of small ducts within heterotopic pancreatic tissue, which can trigger recurrent episodes of acute pancreatitis [20]. In such cases, the condition manifests as GP without evidence of CP in the remaining pancreatic tissue. When the toxic effects of alcohol and smoking impact the ectopic pancreatic tissue, GP may develop along with CP features in the rest of the pancreas [9,21].

There are several mechanisms behind the pancreatic injury induced by alcohol, which leads to CP. It stimulates pancreatic atrophy, increases the viscosity of pancreatic secretions and the formation of proteinaceous plugs in pancreatic ducts, promotes Brunner’s gland hyperplasia, and changes the pancreatic secretion quality, by reducing the citrate concentration, which will further stimulate crystallization in pancreatic juice and will consequently play the role of a nidus for pancreatic stone development [17,22].

Smoking, on the other hand, triggers pancreatic injury by disrupting signal pathways in acinar cells, causing increased intracellular calcium and reduced pancreatic blood flow. The elevation of cytoplasmic calcium will further cause the dysregulation of the cystic fibrosis transmembrane conductance regulator (CFTR), which is involved in chloride and bicarbonate ion transport, leading to decreased fluid secretion and a protein-rich pancreatic juice that promotes premature enzyme activation, damaging acinar cells [23,24]. Smoking also raises endothelin-1 levels, causing vasoconstriction and reduced pancreatic blood flow [24,25]. Additionally, nicotine alters gene expression in the pancreas, affecting trypsinogen regulation [26]. Finally, smoking triggers the activation of the inflammatory cascade and necro-inflammatory processes, eventually leading to fibrosis [24].

The association between alcoholism and tobacco smoking and GP is well known, as well as the male predominance [7,15]. A systematic review, including 8 studies and 335 patients with GP, found a percentage of alcohol abuse of 90% among the included patients and a rate of smoking of 94% [15]. Similarly, in our study, the percentage of alcohol consumers and smokers was high (78.2% and 73.9%, respectively) and the majority of included patients were men (95.6%).

In addition to the alcohol and tobacco consumption, other risk factors responsible for the development of GP are anatomic anomalies such as pancreas divisum, annular pancreas, or ectopic pancreas [8]. One of our patients had an MRI image in favor of annular pancreas, together with the typical imagistic features of GP as duodenal wall thickening, intramural cysts, and pancreatic head enlargement. There was no patient with pancreas divisum in our cohort.

### 4.2. Clinical Features

**Figure 6:**
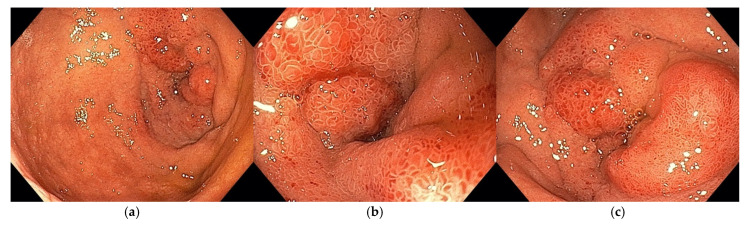
(**a**–**c**) GP with inflammatory stenosis at the level of the upper duodenal genu.

As the inflammation in the groove area can often lead to duodenal stenosis, the clinical manifestations of GP comprise weight loss, upper abdominal pain, postprandial vomiting, and nausea [7,17]. Groove inflammation can create a clinical picture of gastric outlet obstruction (GOO), usually at the level of the upper duodenal genu and second duodenum, but can also occur in the duodenal bulb (Figure 6a–c).

**Figure 7:**
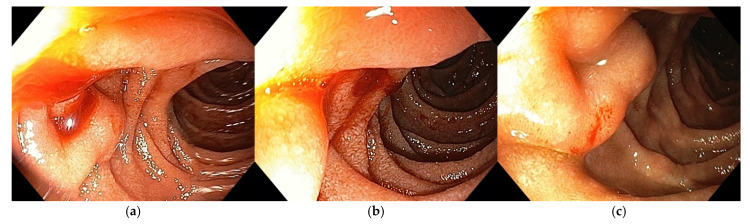
(**a**) Upper GI bleeding in the second portion of the duodenum–edematous, thickened and friable mucosa. (**b**) Small ulcer between the thickened duodenal folds. (**c**) Follow-up endoscopy.

If the inflammation impairs the bile outflow through the CBD, patients can present with cholestasis and jaundice. Our results strengthen these data, as pain was the main symptom in our patients (91.3%), followed by nausea (56.5%) and vomiting (43.5%). Weight loss was observed in approximately one-quarter of the patients in our study. Reports in the literature vary widely, with some studies highlighting a high prevalence of reduction in body weight, while others report findings consistent with our results [6,27]. We also encountered an unusual initial clinical presentation of GP [28] in a patient who presented to the emergency room with upper GI bleeding and melena. The upper endoscopy revealed bleeding from an edematous, thickened and friable mucosa of the second portion of the duodenum, with a small ulcer (Figure 7a,b), for which endoscopic hemostasis was done, with good healing on follow-up examination (Figure 7c).

### 4.3. Endocrine and Exocrine Insufficiency in GP

The interconnectivity between exocrine and endocrine pancreatic disorders has been increasingly explored [29]. It is well established that CP is associated with a greater risk of having DM, as around 23% of patients with CP have a history of type 2 DM at the time of the diagnosis, and an additional 13% are likely to develop it in the following decade [30]. The recognition of pancreatogenic DM, also known as type 3c DM, has led to its reported incidence surpassing that of type 1 DM [31]. However, there is a paucity of data regarding the glycemic abnormalities and DM in patients with GP in the literature. In our study, out of the 23 included patients, only 3 (13%) previously had DM, with none of them being newly diagnosed with DM. Accordingly to studies performed on rodents, islets located in the head of the pancreas possess a greater capacity for insulin synthesis and secretion [32]. Based on this hypothesis, patients with GP might be at an increased risk of developing DM. However, human studies have demonstrated a uniform distribution of islets [33], with a similar density observed in the head and body regions [34]. Additionally, the density of islet is two-fold higher in the tail of the pancreas [34]. This distribution pattern may explain the low rate of DM observed in GP patients in our study.

The hyperglycemia observed in 13 patients (56.5%) is challenging to interpret, as it may represent an adaptive response to metabolic stress and cortisol release during the acute phase of the disease. These, plus the counterregulatory hormones such as glucagon, catecholamine, and growth hormone, together with excessive cytokine release, induce glycemic metabolism modifications, with increased hepatic gluconeogenesis and impaired insulin-mediated glucose uptake into skeletal muscle, leading to hyperglycemia [35,36].

Exocrine pancreatic insufficiency (EPI) in GP can be attributed to the inflammation extending to the pancreatic head and papillary area, which can obstruct normal enzyme secretion. However, an additional contributing factor may be the involvement of the congestive duodenal mucosa, potentially impairing the entero-acinar axis. This disruption could lead to a decreased release of cholecystokinin (CCK) and secretin, key hormones that stimulate pancreatic enzyme secretion [37]. Furthermore, there is a strong association between smoking and EPI, with smoking being an independent risk factor for EPI in CP patients [38]. We remind that the majority of GP patients are smokers. Notably, most patients in our study had an acute presentation, and exocrine insufficiency was not assessed during the acute flare.

### 4.4. Imaging Characteristics

The most important and often most challenging differential diagnosis for GP is groove carcinoma (GC). This diagnosis heavily depends on imaging findings from CT/MRI. The descriptor “tumor-like aspect”, frequently encountered in radiological reports, can be misleading, generating potential confusion with malignancy. In our study, the syntagma “tumor-like aspect” was seen in 39.1% of patients that finally had a diagnosis of GP.

Typically, the CT scan shows a low enhancing hypodense area in the pancreatoduodenal groove, together with a thickening of the duodenal wall and paraduodenal cysts—shown in Figure 6 [11]. In the segmental form, the MPD may be slightly dilated, while in the pure form it appears nondilated. In our study, MPD dilatation was noted in 47.8% of the patients. The CBD may also be dilated, associating mild distension of the intrahepatic biliary system. However, peripancreatic vessels are preserved, with no signs of thrombosis or infiltration even in extensive disease [12]. On MRI, GP is described as a hypointense mass on T1-weighted images and an isointense or mildly hyperintense area on T2-weighted images [11,12]. Kalb et al. found a diagnostic accuracy of 87.2% if using MRI to diagnose GP, when considering three criteria: focal thickening of the second part of the duodenum, abnormally increased enhancement of the descending duodenum, and cystic changes in the area of the dorsal pancreatic duct. If all three of these criteria are fulfilled, the negative predictive value to exclude pancreatic cancer is 92.9% [13]. As presented in the literature, the duodenal wall thickening was also the most frequent pathological feature described in the CT or MRI results of the patients with GP included in our study. Pancreatic head enlargement was observed in more than half of our patients (52.2%), likely associated with an acute exacerbation of CP affecting the groove area and the pancreatic head, which led to hospital admission and imaging evaluation. Even if GP is a form of CP, pancreatic calcifications as a feature of CP were found in less than a third of included patients (30.3%).

The EUS is performed in GP in patients with a high suspicion of malignancy. However, caution is warranted, as the EUS findings evolve over time with disease progression. In the early stages, inflammation predominates, but as the condition advances, fibrosis gradually replaces inflammation, leading to corresponding changes in the sonographic appearance. On EUS, GP is initially described as a hypoechoic or heterogeneous mass along with the second part of the duodenum, together with duodenal wall thickening with loss of stratification, periduodenal and intramural fluid collections, narrowing of the duodenal lumen, and stenosis of the common bile duct or pancreatic duct [11,12]. In late GP, patients have a hyperechoic duodenal wall and a hyperechoic dorso-cranial part of the pancreatic head. The changes are due to myoadenomatoid proliferation and fibrosis of the adjacent pancreas. The duodenal wall is thick because of the hypertrophy of the submucosal layer due to hyperplasia of the Brunner glands. Plus, in late stages, the hypoechoic area within the groove area may be absent [11]. Duodenal wall thickening (68.4%) and paraduodenal and duodenal wall cysts (52.6%) were the main features described in the EUS report of the patients included in our study, while the “tumor-like aspect” was described in 31.6% of patients. As for pancreatic calcifications, they were described in 36.8% of patients. We note that even if they are a well-known feature of CP, frequently associated with alcohol use, pancreatic calcifications are less described in GP [6,39]. They are often a hallmark of chronicity, correlated with extensive fibrosis and a greater risk of duodenal stenosis [40,41]. Thus, their identification on imaging can provide valuable insights about the length of the disease, local complications, and prognosis, as their presence may influence the treatment approaches toward invasive interventions.

When comparing the characteristics of GP and GC, features in favor of GP are as follows: presence of cystic lesions in the groove/duodenal wall, a regular and smooth tapering of the common bile duct or pancreatic duct, duodenal wall thickening, and leftward luxation of gastroduodenal artery. Conversely, in favor of GC are the following: the absence of alcohol and tobacco consumption, the absence of cysts in the duodenal wall, a blunt caliber change of the common bile duct, vascular encasement, jaundice, and high CA19-9 levels [42,43,44,45]. Another feature in favor of GC would be the abrupt pancreatic duct cutoff sign and consequent parenchymal atrophy [46].

**Figure 8:**
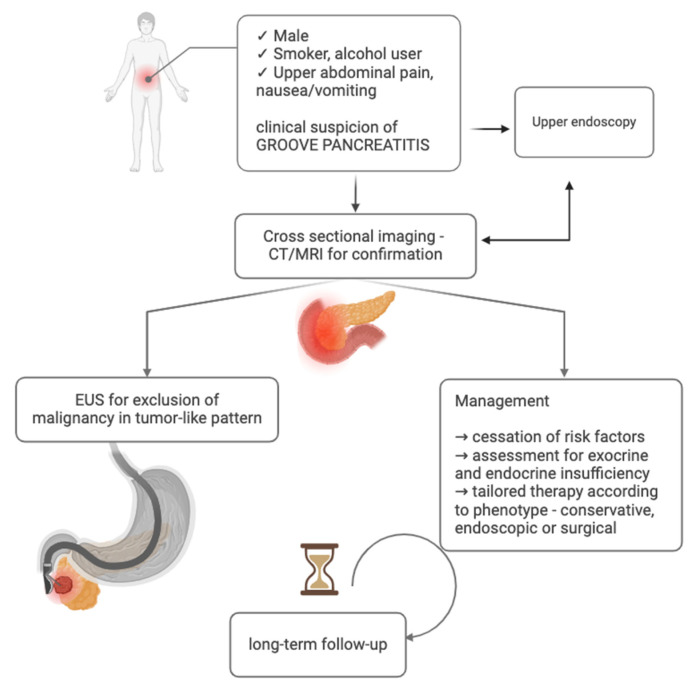
Diagnostic workup in GP (created with BioRender.com).

In light of the above-mentioned clinical, imaging, and endoscopic data, we propose the following diagnostic workup for patients with suspected GP—shown in Figure 8.

### 4.5. Therapeutical Approach

**Figure 9:**
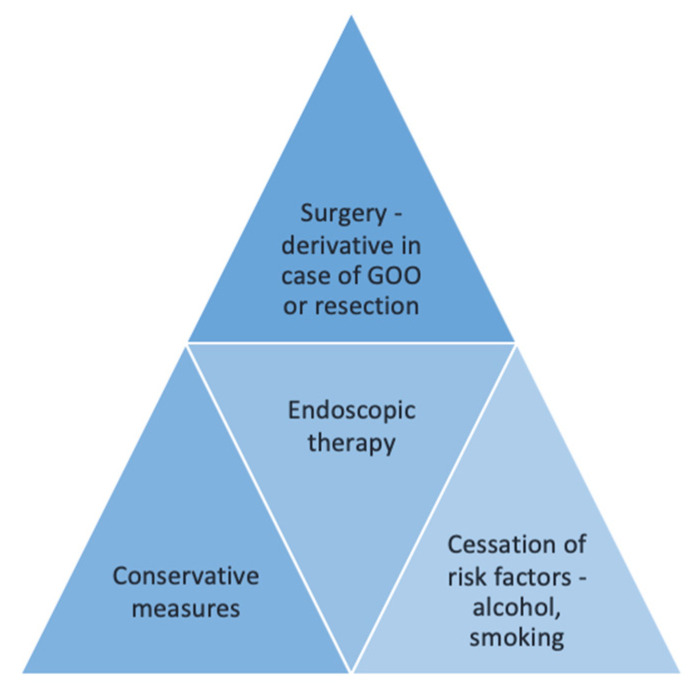
Therapeutical approach in GP.

Once the malignancy is excluded, a step-up approach should be considered, starting with a conservative attitude and continuing with endoscopic, followed by surgical therapy if no response—shown in Figure 9. However, this step-up approach might be reconsidered based on the inflammatory versus fibrotic pattern of disease, with the latter form of GP being less responsive to conservative measures.

**Figure 10:**
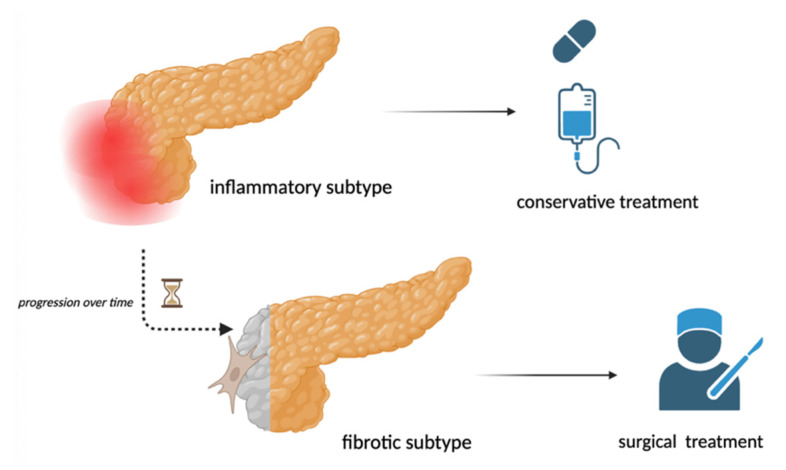
Therapeutic approaches in different subtypes of GP (created with BioRender.com).

Thus, we propose that GP could be classified into two distinct phenotypes—an inflammatory subtype and a fibrotic subtype—each requiring a tailored therapeutic approach. Conservative treatment might be more effective during the inflammatory stage of GP, while patients with long-standing disease and fibrotic complications could benefit from earlier referral to interventional procedures (Figure 10). It is worth mentioning that endoscopic treatment is proper only in the early stages of the disease and can be difficult in the later stages, because of duodenal scarring and stenosis, difficult duodenal intubation, and restricted access to major or minor papillae. Due to the low number of patients requiring interventions, we could not draw definitive correlations between disease duration and need for endoscopic/surgical therapies.

Among the pitfalls in diagnosis, there is the potential responsiveness of GP to steroids [47], which has been also used as a therapeutic trial in differentiating autoimmune pancreatitis and pancreatic cancer [48]. Lymphoma involving the duodenum might also show a response to steroids [49]. Moreover, inflammation in the setting of AP may mask an underlying cancer, and we should keep in mind that patients with a pancreatitis flare, especially those with underlying CP, have a greater risk for pancreatic malignancy [50]. However, the steroid diagnostic trial is generally not recommended and is reserved exclusively for selected patients who have had negative results across the complete diagnostic workup, including EUS-guided tissue acquisition [48,51].

In our study, most of the patients were treated conservatively, including pancreatic rest, pain control and cessation of smoking or alcohol consumption. Of note, long-acting somatostatin analogs are not reimbursed in Romania for GP, and none of the included patients could benefit from it. Endoscopic therapy was performed in only 2 patients, whereas 4 patients underwent surgical treatment. The limited number of endoscopically treated cases may be attributed to the low availability of interventional endoscopic treatment at the time of diagnosis. The most frequent surgical intervention for GP is pancreatoduodenectomy. Other surgical techniques are pancreas-preserving duodenal resection, duodenum-preserving pancreatic head resection, gastroenterostomy, pseudocyst drainage, and hepatico-jejunostomy [15].

Regarding treatment efficiency, in the systematic review by Kager et al., the greatest success in obtaining complete pain relief was noted in patients that underwent surgical treatment (79%) [15]. However, the conservative and endoscopic treatments are not to be neglected, as they have considerable percentages of success in relieving the pain (50% and 57% respectively), especially in the context of elevated mortality and morbidity of surgical interventions such as pancreatoduodenectomy [15]. In the same study, the percentage of reported complications was almost double in surgical patients (20%) compared to those treated conservatively (11%), while patients treated endoscopically had a complication percentage of 13% [15]. In patients who underwent pancreatectomy, the most common pathological findings were cystic lesions in the duodenal wall, Brunner gland hyperplasia, dilation of Santorini’s duct, and protein plaques in the pancreatic duct [17].

As EUS-guided therapies and ERCP gain more and more ground ahead of surgical approaches for drainage and derivative purposes, it is expected that surgery will remain a therapeutic option mainly for resections. Moreover, as awareness of GP continues to grow among physicians and a deeper understanding of its risk factors, progression, and efficacy of conservative and endoscopic management, treatment strategies are increasingly shifting towards less invasive approaches.

Regardless of the therapeutical approach, the need for follow-up is essential in all patients, as GP is a form of CP that carries a long-term risk of progressing to PC [50].

### 4.6. Study Limitations

One of the most important limitations of our study is the heterogeneity of the imaging written reports, as a result of the different levels of expertise among the radiologists. Considering the rarity of GP, it is plausible that radiologists with less experience may not recognize its characteristics, which could further contribute to variability and inconsistencies in the reports. Another limitation of the study is that the majority of patients were evaluated during their presentation to the emergency room, likely for an acute exacerbation of their chronic condition. Consequently, the clinical manifestations, biological findings, and imaging characteristics predominantly reflected the acute phase in most cases. The heterogenous follow-up period is another limitation to be taken into consideration. Even if it is a rare condition, the small number of included patients is one of the most important limitations, which should encourage further studies within multicentric collaboration. Because of small sample size in each GP subgroup, we were unable to detect statistically significant differences. The delineation of the clinical phenotype and outcomes in pure GP compared to segmental and diffuse remains to be established.

## 5. Conclusions

Our study reinforces the understanding that GP predominantly affects men with a history of chronic alcohol abuse and smoking. The study highlights the clinical, imaging, and management differences between tumor-like and non-tumor-like patterns of GP. The tumor-like pattern, observed in around a third of cases, presents a significant diagnostic challenge due to its resemblance to pancreatic malignancy. Being a rare disease, a standardized diagnostic approach is essential. A possible delineation between an inflammatory subtype and a fibrotic subtype of GP according to the duration and evolution of the disease could orientate the physician toward an appropriate individualized therapeutic approach.

## Figures and Tables

**Table 1:** Patients’ characteristics in the subgroups with and without tumor-like appearance.

	*Tumor-Like*	*Non-Tumor-Like*
*Number of patients*	11	12
*Demographics and medical history*		
*Age (years)*	54.0 (45.5–57.5)	56.5 (48.75–59.75)
*Male gender*	10 (90.9)	12 (100)
*Alcohol use*	10 (90.9)	8 (66.7)
*Smokers*	8 (72.7)	9 (75)
*History of DM*	1 (9.1)	2 (16.7)
*Symptoms*		
*Pain*	11 (100)	10 (83.3)
*Vomiting*	6 (54.5)	4 (33.3)
*Nausea*	6 (54.5)	7 (58.3)
*Jaundice*	0 (0.0)	1 (8.3)
*Weight loss*	2 (18.2)	4 (33.3)
*Upper GI bleeding*	1 (9.1)	0 (0.0)
*Laboratory workup*		
*Glycemia (mg/dL)*	110.5 (99.5–127.5)	99.5 (84.75–127.0)
*CA19-9 (U/mL)*	7.05 (3.78–22.57)	14.46 (2.85–67.62)
*Albumin (g/dL)*	4.34 (3.72–4.8)	4.0 (3.7–5.0)
*Treatment*		
*Conservative*	8 (72.7)	9 (75)
*Endoscopic*	1 (9.1)	1 (8.3)
*Surgical*	2 (18.2)	2 (16.7)
*Mortality, n (%)*	3 (27.3)	1 (8.3)
*Cardiovascular diseases*	2 (18.2%)	0 (0.0)
*Severe alcoholic hepatitis*	1 (9.1%)	0 (0.0)
*Pulmonary malignancy*	0 (0.0)	1 (8.3)

**Table 2:** Imaging findings in patients with GP.

Pathological Findings at CT/MRI n (%)	Total, n = 23	Tumor-Like Pattern, n = 9	Non-Tumor-Like Pattern, n = 14
Pancreatic head enlargement	12 (52.2)	5 (55.6)	7 (50)
Duodenal wall thickening	14 (60.9)	9 (100)	5 (35.7)
Intramural and paraduodenal cysts	12 (52.2)	4 (44.4)	8 (57.1)
CBD narrowing	5 (21.7)	3 (33.3)	2 (14.3)
MPD dilatation	11 (47.8)	6 (66.7)	5 (35.7)
Pancreatic calcifications	7 (30.4)	4 (44.4)	3 (21.4)
Tumor-like aspect	9 (39.1)	9 (100)	0 (0)

**Table 3:** EUS findings in patients with GP.

Pathological Findings at EUS n (%)	Patients, n = 19	Tumor-Like Pattern, n = 7	Non-Tumor-Like Pattern, n = 12
CBD thickening	2 (10.5)	1 (14.3)	1 (8.3)
CBD dilatation	3 (15.8)	0 (0)	3 (25)
Pancreatic head enlargement	5 (26.3)	4 (57.1)	1 (8.3)
Duodenal wall thickening	13 (68.4)	7 (100)	6 (50)
Intramural and paraduodenal cysts	10 (52.6)	4 (57.1)	6 (50)
Pancreatic calcifications	7 (36.8)	5 (71.4)	2 (16.6)
Tumor-like aspect	6 (31.6)	6 (85.7)	0 (0)

**Table 4:** Treatment approaches in GP.

*Treatment Type* *n (%)*	*Total,* *n = 23*	*Tumor-Like Pattern, n = 11*	*Non-Tumor-Like Pattern, n = 12*
Conservative only	17 (73.9)	8 (72.7)	9 (75)
Endoscopic therapy	2 (8.7)	1 (9.1)	1 (8.3)
*Cyst aspiration/drainage*	*1*	*1*	*0*
*Duodenal dilation*	*0*	*0*	*0*
*CBD stenting*	*1*	*0*	*1*
*PD stenting*	*0*	*0*	*0*
*EUS-GE*	*0*	*0*	*0*
Surgery	4 (17.4)	2 (18.2)	2 (16.6)
*Gastrojejunostomy*	*3*	*2*	*1*
*Pancreaticoduodenectomy*	*0*	*0*	*0*
*Pseudocystojejunostomy*	*1*	*0*	*1*

## Data Availability

Datasets are available from the corresponding author.
